# The Next Challenges of Vestibular Implantation in Humans

**DOI:** 10.1007/s10162-023-00906-1

**Published:** 2023-07-29

**Authors:** Joost Johannes Antonius Stultiens, Richard F. Lewis, James O. Phillips, Anissa Boutabla, Charles C. Della Santina, Rudolf Glueckert, Raymond van de Berg

**Affiliations:** 1 Department of Otorhinolaryngology & Head and Neck Surgery, School for Mental Health and Neuroscience, Faculty of Health Medicine and Life Sciences, Maastricht University Medical Center, P. Debyelaan 25, Maastricht, 6202 AZ The Netherlands; 2 Department of Otolaryngology and Neurology, Harvard Medical School, Boston, MA USA; 3 Department of Otolaryngology, University of Washington, Seattle, WA USA; 4 Department of Otorhinolaryngology & Head and Neck Surgery, Department of Clinical Neurosciences, Geneva University Hospitals, Geneva, Switzerland; 5 Department of Biomedical Engineering and Department of Otolaryngology - Head & Neck Surgery, Johns Hopkins School of Medicine, Baltimore, MD USA; 6 Department of Otolaryngology, Medical University of Innsbruck, Innsbruck, Austria

**Keywords:** Vestibulopathy, Bilateral vestibulopathy, Vestibular implant, Vestibular neuroprosthesis, Humans

## Abstract

Patients with bilateral vestibulopathy suffer from a variety of complaints, leading to a high individual and social burden. Available treatments aim to alleviate the impact of this loss and improve compensatory strategies. Early experiments with electrical stimulation of the vestibular nerve in combination with knowledge gained by cochlear implant research, have inspired the development of a vestibular neuroprosthesis that can provide the missing vestibular input. The feasibility of this concept was first demonstrated in animals and later in humans. Currently, several research groups around the world are investigating prototype vestibular implants, in the form of vestibular implants as well as combined cochlear and vestibular implants. The aim of this review is to convey the presentations and discussions from the identically named symposium that was held during the 2021 MidWinter Meeting of the Association for Research in Otolaryngology, with researchers involved in the development of vestibular implants targeting the ampullary nerves. Substantial advancements in the development have been made. Yet, research and development processes face several challenges to improve this neuroprosthesis. These include, but are not limited to, optimization of the electrical stimulation profile, refining the surgical implantation procedure, preserving residual labyrinthine functions including hearing, as well as gaining regulatory approval and establishing a clinical care infrastructure similar to what exists for cochlear implants. It is believed by the authors that overcoming these challenges will accelerate the development and increase the impact of a clinically applicable vestibular implant.

## Introduction

Over the past two decades, the feasibility of a vestibular implant was demonstrated. This neuroprosthesis can provide motion information to the vestibular nerve through implanted electrodes. It was designed to aid patients with bilateral vestibulopathy (also called bilateral vestibular hypofunction), a condition that can lead to a severe impairment of quality of life due to physical symptoms, such as imbalance and oscillopsia, as well as a negative impact on cognition and mood [1, 2]. Etiologies vary, but patients are most frequently diagnosed with genetic disorders, Menière’s disease, ototoxicity infectious diseases, and neurodegenerative diseases. However, in approximately, a third of the patients the etiology remains idiopathic [3]. Vestibular hypofunction leads to a high individual and socioeconomic burden and no cure is available yet [2]. Vestibular rehabilitation may improve some complaints, but this gain is limited and insufficient for many individuals [4]. An estimated 1.8 million adults worldwide live with disability and symptoms consistent with chronic bilateral vestibulopathy [5]. Solutions to alleviate the impact of this condition are desired, for individual patients and for society.

**Fig. 1:**
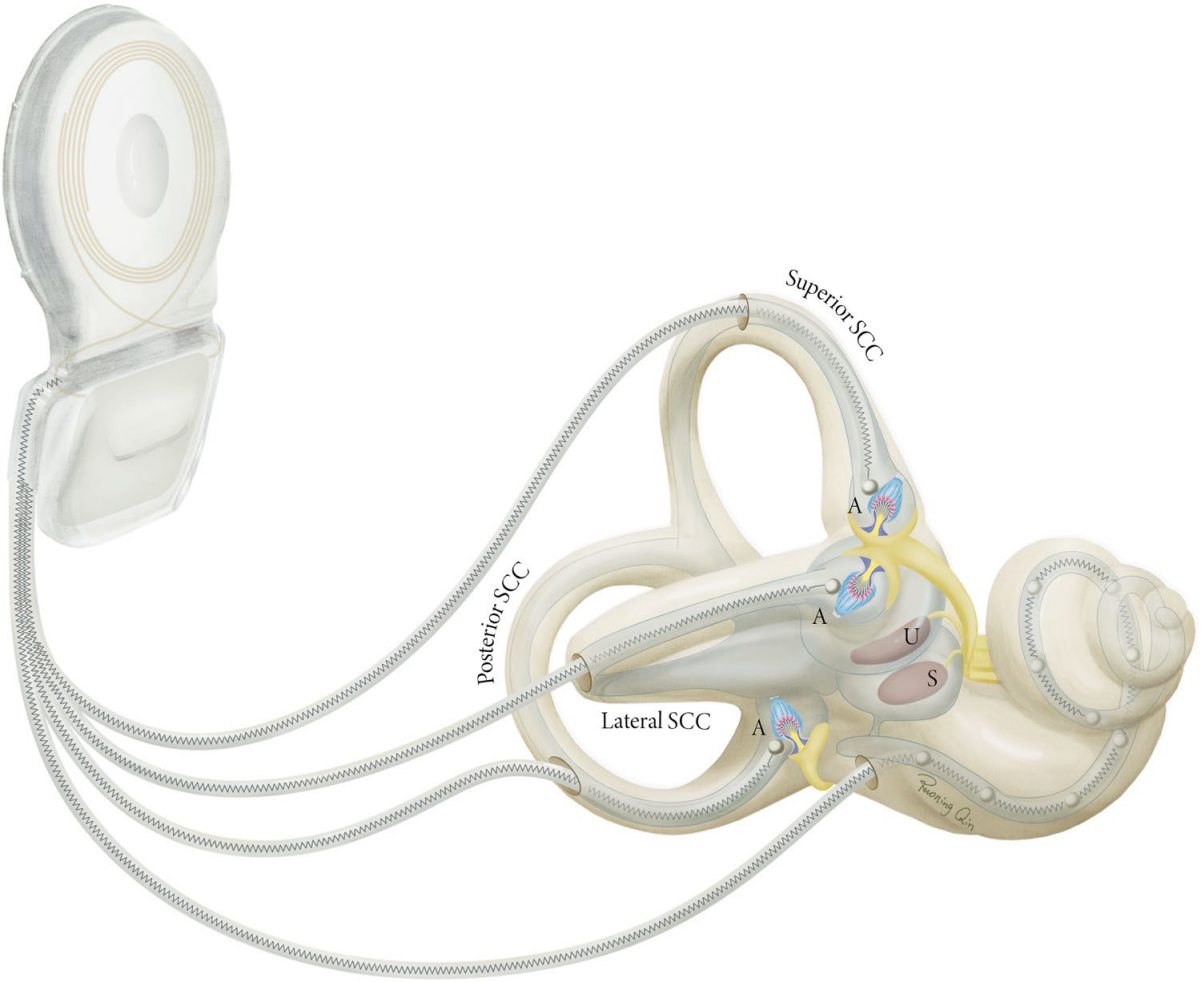
The vestibular implant, combined with a cochlear lead (i.e., a vestibulocochlear implant), in relation to the bony and membranous labyrinth of the inner ear. The electrodes implanted through fenestrations in the bony semicircular canals stimulate the terminal afferent nerve fibers from the ampullae. SCC, semicircular canal; A, ampulla; U, utricle; S, saccule. *Illustration made by Ruoning Qin*

The vestibular system consists of several structures bilaterally present in the skull. The vestibular organs are located in both vestibular labyrinths (Fig. 1), protected within the temporal bone, where they supply the central vestibular system with sensory information through the eighth cranial nerve. Angular accelerations of the head are mainly sensed by the semicircular canals and linear accelerations mainly by the otolith organs. In daily life, most head movements will often result in activation of all sensors. The three semicircular canals (Fig. 1SCC) have a widening just before their utricular opening in the vestibule; the ampulla (Fig. 1A). Here, the crista ampullaris, with a covering gelatinous mass, the cupula, embeds the hair cells, which are connected to the terminal vestibular nerve fibers to form the ampullary nerve of each canal. The three semicircular canals of each ear are oriented almost orthogonally to each other. Consequently, the six semicircular canals have complementary and opposing optimal sensitivity and together can sense movements in all directions [6]. The utricle (Fig. 1U) and saccule (Fig. 1S), the otolith organs, are located in the vestibule. Their sensory epithelium, the macula, is oriented orthogonally for both organs, with the utricular macula being mainly in the horizontal plane and the saccular macula mainly in the vertical plane. Due to the inertia of mass, these organs are sensitive to linear accelerations, as well as rotations/centrifugation and tilt [6].

Research on electrical stimulation of the vestibular system began many years ago. In 1874, electrical stimulation targeting individual semicircular canals of a pigeon was performed to investigate the functioning of these organs, showing that this stimulation resulted in a (head) nystagmus in the plane of the stimulated canal [7, 8]. In the 1960s, a seminal series of animal experiments regarding eye movement reflexes was conducted [9–11]. Since then, much research that was primarily performed to understand the cellular and systems level physiology of the vestibular system has also provided a basis for prosthetic electrical stimulation of the vestibular labyrinth. This includes research on how electrical currents excite vestibular primary afferent neuron activity [12, 13] and on the spatiotemporal dynamics [14], directional plasticity [15, 16], and rotational kinematics of the vestibulo-ocular reflex (VOR). Those lines of research converged with decades of cochlear implantation technology development to lead to the idea to develop a vestibular neuroprosthesis to aid patients suffering from vestibulopathy. By 2000, a single-channel prototype for use in guinea pigs had been built and studied [17, 18]. Extensions of that concept from rodents to nonhuman primates, from acute to chronic stimulation, from single-channel to multi-channel stimulators, and from a single laboratory to a small but productive international community ultimately established compelling preclinical results justifying clinical trials of vestibular implantation [18–29].

After an initial foray into intraoperative stimulation of the human vestibular nerve [30], vestibular implant prototypes with three electrode leads were developed to stimulate the ampullary nerve of each semicircular canal [31–33]. These electrodes can be inserted either extralabyrinthine, directly adjacent to the ampullary nerve canals, or intralabyrinthine with each electrode lead inserted in the semicircular canal with the stimulating electrode in the ampulla near the ampullary nerves. Motion information from gyroscopes and accelerometers that can be implanted under the skin or magnetically connected, is then used to stimulate these vestibular afferents. In this way, the patient’s nervous system obtains the missing information to incorporate it in the various bodily functions associated with vestibular input.

Several research groups around the world are working on development of a vestibular neuroprosthesis. Findings in animal research have been translated to human subject trials. The aim of this review is to illustrate the advancements made to develop a vestibular implant and to confer the next challenges for this research field, as discussed during the 44^th^ Annual MidWinter Meeting of the Association for Research in Otolaryngology, 2021 [34].

### Animal Research

The feasibility of a vestibular implant has been investigated in animal models by various research groups around the world. The symposium highlighted the advancements made in animal studies by the Massachusetts Eye and Ear/Harvard Medical School group.

The primary findings and their potential clinical and scientific implications [35] are summarized below.

One of the first steps was to implant an electrode in the lateral semicircular canal of guinea pigs, in order to examine how stimulation parameters affect the resulting eye movements [17, 18]. Biphasic, charge-balanced current pulses were used as the primary stimulation unit in these and all subsequent studies, and the frequency of these pulses and their amplitude (current) were varied independently. It was found that the nystagmus rate correlated with stimulation rate and amplitude in a fairly linear manner over a wide range but eventually saturated [36]. Related findings were that turning on and off a tonic rate of stimulation provoked a rapid nystagmus response, the former due to the sudden tone imbalance generated by the high rate of tonic stimulation (e.g., 200 pulses-per-sec) and the latter as an “after-effect” which indicates that the brain adapted to the stimulation. When stimulation was repeatedly turned on and off, however, the nystagmus response attenuated with these repeated on–off transitions. While this change could be due to habituation (defined as a non-specific (non-associative) reduction in the brain’s sensitivity to a sensory stimulus after repeated or prolonged exposure) or adaptation (defined as a specific (associative) change in central processing that serves to reduce the magnitude of a sensorimotor error), evidence supporting the latter was found in experiments where the amount of stimulation between two groups of animals was kept constant but the dynamics with which it was presented varied [23].

Following these static experiments in rodents, dynamic VOR responses were investigated in squirrel monkeys. The lateral canals on both sides were inactivated with a plugging procedure and a one-dimensional canal prosthesis was then utilized, with the sensor aligned with the lateral canals and the electrode implanted in one lateral canal ampulla [19]. With this approach, it was found that a considerable fraction of the normal yaw-axis VOR could be recovered with the prosthesis and that the VOR response appeared to use visual feedback to adapt its amplitude and rotational axis [19, 22]. This VOR response did have a very short time constant, however, and this did not change over time. The animals received vestibular implant stimulation continuously in their cage for up to 1 year, and thus the effects of chronic stimulation could be investigated. The short-time constant suggests that velocity storage may not be engaged by prosthetic stimulation. Since velocity storage is a critical component of central vestibular processing and is the substrate that synthesizes canal (angular velocity) and otolith (gravito-inertial acceleration) signals received from the labyrinth, the ability of a canal vestibular implant to engage velocity storage was investigated directly in an acute stimulation experiment in squirrel monkeys [37]. One lateral canal was stimulated with the animal upright or tilted in roll, and it was found that when tilted, the induced VOR response attenuated more rapidly than when upright (e.g., “dumping” occurred). Furthermore, the eye’s rotational axis shifted to align with gravity (e.g., “spatial orientation” of the VOR occurred). Both of these eye movement responses require velocity storage. Hence, these experiments showed that the prosthetic angular velocity information was indeed synthesized in the brain with otolith cues through the velocity storage mechanism.

The following experiments were performed in rhesus monkeys and focused on psychophysics as well as eye movements, utilizing a three-dimensional semicircular canal prosthesis. Firstly, an approach was developed to have the monkeys indicate their perceived direction of upright (e.g., the direction aligned with gravity) using a task similar to the subjective visual vertical (SVV) test that is often used in human subjects. It was shown that the monkeys perceived tilt in a manner that recapitulated many features of the human response, for example, the illusion of tilt during centrifugation [38]. The initial prosthesis–perception studies were acute in nature and involved stimulating one posterior canal electrically while the animal was upright, stationary, and performing the SVV task. It was found that stimulation of one posterior canal induced an illusion of tilt (as evidenced by the SVV responses) towards the stimulated ear, indicating that the angular velocity information provided by the canal input altered the animal’s perception of head position relative to gravity [39]. Following these acute perceptual studies, chronic experiments in rhesus monkeys investigated if the three-dimensional vestibular implant could improve the perception of head orientation in space during dynamic tilts after the normal inner ear function was ablated [40]. Normal monkeys could accurately perceive the direction of gravity during dynamic roll tilts, but this capability was severely diminished following bilateral vestibular ablation. When the unilateral three-dimensional vestibular implant was chronically activated, however, the perception of gravity improved, indicating that the animals utilized prosthetic angular velocity cues to improve their perception of head position in space relative to gravity.

Three-dimensional VOR measurements showed that the vestibular implant can generate compensatory three-dimensional VOR responses. Furthermore, the VOR threshold for yaw-axis rotations, i.e., the smallest angular motion that elicits a corrective VOR slow phase, was measured. These responses are of particular interest because the threshold is related to the signal-to-noise ratio in the brain by signal detection theory. It was found that normal monkeys had VOR thresholds for the yaw axis similar to humans, that the threshold increased dramatically after vestibular ablation, and then decreased when the vestibular implant was activated. Over time, the threshold became slowly smaller, which implies that the brain’s ability to segregate the vestibular signal from the noise was improving through an adaptive mechanism.

Most recently, balance was studied in these rhesus monkeys using a balance platform that was designed to allow them to maintain their normal quadrupedal stance. Visual cues (e.g., lights on or off) and proprioceptive cues (standing on thin rubber or thick compliant foam) were manipulated and the platform was tilted in a complex manner (using a pseudo-random ternary sequency or PRTS paradigm) to examine how their balance was perturbed by motion of the support surface [41, 42]. Finally, the monkeys were trained to turn their heads in response to visual targets, so that the influence of head motion on balance could be examined [43]. These approaches enabled studying the effects of vestibular ablation during quiet stance, rotation of the support surface, and during head turns, and identified a number of pathologic changes in postural control after vestibular ablation [41–43]. The head turn paradigm was also utilized with the vestibular implant and showed that the animal’s trunk stability improved when prosthetic 3D angular velocity information was provided [43].

In sum, these animal studies investigated how semicircular canal information provided by a one or three-dimensional prosthesis affects VOR, perception, and postural responses and showed that after vestibular ablation all of these behaviors are degraded but that activating the vestibular implant in these animals improves all three of these vestibular-mediated behaviors. These results also show that the brain synthesizes the vestibular implant angular velocity information with visual feedback, otolith information, and activates the brain’s velocity storage network. Finally, the brain can temporally integrate the three-dimensional angular velocity information provided by the vestibular implant to estimate head position in space, a crucial capability if the prosthesis is to improve spatial orientation and postural control in patients with severe vestibular damage. This work helped to provide the preclinical foundation [44] that supported the development of vestibular implant studies in human subjects, while simultaneously providing scientific information about normal vestibular processing in the brain.

### Human Subject Research

The knowledge gained by the experiments with animals, paved the way for human vestibular implant research. Currently, several research groups around the world are conducting clinical investigations in humans to accelerate the translation to the clinic to aid patients with (bilateral) vestibulopathy. The devices currently under investigation are based on the concept of the cochlear implant, the analog for auditory stimulation, and are designed to directly stimulate the vestibular nerve branches, bypassing the damaged vestibular organs. In the past two decades, significant advancements have been gained in human subject research.

In 2007, it was reported that electrical stimulation of the posterior ampullary nerves elicits nystagmic eye movement responses [30]. Later, this was also shown for the lateral and superior ampullary nerves [45]. This encouraged the development of prototype vestibular implants to stimulate first one [46] and later all three ipsilateral ampullary nerves [31, 33, 47, 48]. The first experiments showed that the baseline electrical activity of these nerves could be modulated to generate bidirectional, smooth eye movements and that adaptation to the electrical vestibular stimulation occurs, similar to or even faster than was found in animals [36, 46]. After coupling the internal part with motion sensors, electrical stimulation in response to head movement could be investigated and (partial) restoration of VOR was demonstrated at low-, mid- [31], and later also high frequencies [49]. It was, however, almost absent at the low frequencies [31], but started to grow from 0.5 Hz [50]. The elicited eye movements were predominantly directed in the plane of the stimulated canal [31, 33, 47]. Functional advancement could also be achieved, as shown by the improved dynamic visual acuity test, which measures the ability to read during walking on a treadmill [51].

The early human subject trials focused on the effect of vestibular stimulation on eye movements, in order to achieve gaze-stabilization functionality in a clinical device. However, this is not the only function of the vestibular system. Postural control, or balance, has a significant impact on quality of life, but the specific contribution of the vestibular input is difficult to measure due to the many organs and sensors that are responsible. However, early trials already showed that postural responses can be elicited by the vestibular stimulation, as was initially shown by platform testing [47]. Later, electrically elicited cervical vestibular evoked myogenic potentials and other measures of postural response were also recorded [52, 53]. A recent trial included home use of a vestibular implant and enabled evaluation of posture and gait after 6 months and 1 year of using a vestibular implant, which generally also showed improvement still after 1 year [53]. Of the main vestibular functions, the effect of the vestibular implant on spatial orientation and/or perception of movement may be most difficult to assess.

## Next Challenges

The feasibility of a vestibular implant was demonstrated in an investigational setting in recent years. There are, however, still many challenges left before the step to a clinically available implant can be made, and even then many challenges will remain to provide the best vestibular stimulation. Here, we will discuss the next steps for vestibular implantation research towards a clinical vestibular implant delivering optimal stimulation to aid patients suffering from vestibular loss.

### Providing Optimal Stimulation

Many factors may influence the feasibility and the result of electrical stimulation. This includes, among others, the etiology, which might impact nerve integrity, the surgical placement (see section below), and the electrical stimulation profile. The electrical stimulation profile (i.e., phase duration, pulse rate, baseline level, modulation depth) affects the velocity of the electrically evoked VOR (eVOR) and the intensity of electrically evoked vestibular percepts [54]. Shorter phase durations and, to a lesser extent, slower pulse rates maximized the electrical dynamic range available to elicit a wider range of vestibular perceptual intensities. Interestingly, however, results showed that larger dynamic ranges did not result in higher velocities of the eVOR. Instead, it was shown that current modulation depth was the key factor with the most consistent impact on the eVOR. These findings suggest that stimulation parameters should be carefully chosen to maximize the range over which currents can be modulated. In a subsequent experiment, this investigation was extended to the simultaneous evaluation of the three main vestibular pathways: the eVOR, the vestibulo-collic reflex pathway (eVCR), and the vestibulo-thalamo-cortical pathway (VTC) responsible of the conscious vestibular percepts. These experiments demonstrated the feasibility and pertinence of the idea of simultaneously recording the responses of three different vestibular pathways. Moreover, results showed that the latencies of the electrically evoked vestibular reflexes were in line with those reported in clinical settings, using sound and motion stimuli. Finally, the amplitude of the vestibular reflexes (eVOR and eVCR) did not seem to be as sensitive to electrical stimulation as the VTC pathway [55]. Understanding the specificity of each pathway is important to achieve specific control and selective activation but will also be of great help to decipher the contribution of each pathway for the global rehabilitation process.

Since the ampullary nerves, and consequently the electrodes, are located close to each other, current from one stimulating electrode intended to spread to the ampullary nerves of the corresponding canal, can also spread to the ampullary nerves of another semicircular canal or to the otololith afferents or cochlear nerve. Consequently, current spread is both a necessary feature of the electrical stimuli delivered by an electrode of a vestibular prosthesis, and also a significant limitation. Electrical activation of human ampullar nerves produces slow phase VOR eye velocities that are often well below the maximal response that would be elicited by natural stimulation of a normal intact human semicircular canal [33, 48, 56]. Increasing the current amplitude of the stimulation increases the elicited eye velocity and the peripherally recorded compound action potential, but the maximally elicited velocities are typically low [21, 56]. This suggests that not all of the afferent fibers of a given nerve are activated by the electrical stimulus [21, 56]. Furthermore, over months of repeated activation, the elicited slow phase eye velocities typically decrease in response to a given current amplitude and pulse frequency, suggesting that fewer and fewer fibers are being activated by the same stimulus over time [56, 57]. Animal studies have shown that central adaptation can significantly compensate for these limitations [26, 58, 59]. However, to obtain useful slow phase eye velocities in humans, typically the current amplitude levels must be increased to the point where a second feature of current spread is observed; i.e., activation of both the targeted nerve and adjacent neural elements. Virtually all studies of human vestibular electrical stimulation suggest the presence of current spread either to adjacent semicircular canals or to the nearby otolith organs. For canal stimulation, this is evidenced by slow phase velocity that is not in the plane of the stimulated canal (e.g., [33, 46, 56]). Further evidence is that with increasing current amplitude, the magnitude of the off direction response typically increases [21, 56]. The utricle is situated such that it is often one of the first non-targeted organs to be activated by current spread of stimulation from the semicircular canals [60]. However, such interactions are not limited to vestibular end organs. Interleaved stimulation of cochlear electrode sites and semicircular canal electrode sites produce interactions modulating the effect of targeted stimulation of one modality on the other [61]. High current levels also activate the facial nerve in vestibular implants and cochlear implants, setting an upper limit to the therapeutic current amplitude [62]. These effects can be somewhat reduced by changing the location of the reference electrode [60]. Still, the optimization of the electrical stimulus to elicit a robust and long-lasting response, while at the same time minimizing off direction or cross modal responses, is one of the remaining challenges in the development of this technology.

Additionally, the vestibular implant prototypes described in this article deliver electrical current close to the ampullary nerves, to stimulate these three ampullary nerves that carry information from angular head accelerations. Next to these three vestibular sensors, each inner ear also contains two otoliths, the utricle and saccule, that are believed to transfer information from linear head accelerations. Utilizing the abovementioned current spread, the current vestibular implant prototypes could possibly also intentionally stimulate these otolith organs encoding linear accelerations. Alternatively, the otoliths could be electrically stimulated with implanted electrodes close to these nerve afferents. Currently, also vestibular implant prototypes to specifically target the otoliths are under investigation [63, 64]. The investigations in humans involve implanting an electrode in the vestibule and subsequently stimulating this area using a constant train of high-frequency electrical pulses. Preliminary results suggested that this led to improved postural ability and gait performance [63]. Nevertheless, this approach poses similar (e.g., cross-excitation/target specificity, hearing preservation) as well as entirely distinct (e.g., surgical approach, response to angular movements) challenges.

### Surgical Implantation

In contrast to auditory nerve afferents which are located along the cochlea, the vestibular nerve afferents are located at specific sites, viz. the three semicircular canal ampullae and the two otolith organs [65]. Consequently, this limits the spots for vestibular stimulation. As stated above, essentially two surgical approaches for vestibular stimulation of the ampullary nerves were developed: an extralabyrinthine approach and an intralabyrinthine approach. The former technique is surgically more challenging and entails a risk of damage to the facial nerve and hearing, and the risk of not reaching all ampullary nerves in some patients. Most current research therefore focuses on the intralabyrinthine technique in which fenestrations are made in the semicircular canals and electrodes are inserted towards the ampullae. The goal is to place the electrodes near the semicircular canal cristae, since this seems to provide the best stimulation, in the vicinity of the ampullary nerves [60]. Besides, as above-mentioned, current spread can impact the selectivity of stimulation. Yet, correct electrode placement is challenging.

Drilling a fenestration very close to the semicircular canal ampulla in order to place the electrodes under microscopic sight might increase the chance of vestibular and auditory damage [66]. Therefore, a “blind” insertion is often carried out through a fenestration at a spot further from the ampulla. Imaging techniques such as high-frequency ultrasound or optical coherence tomography have a high resolution, but seem to lack the ability to penetrate the covering (skeletonized) bone to visualize the ampullae. Fluoroscopy-guided vestibular implantation in human cadaver heads showed substantial improvement of electrode placement, although visualization of the semicircular canal ampullae is still challenging [67]. Intraoperative measurement of objective vestibular responses such as VORs or electrically evoked compound action potentials of the vestibular nerve (veCAPs) could possibly relate the position to the stimulation effect and so indicate the optimal placement. However, intraoperative VOR measurements are influenced by anesthetics and are not always reliably obtained during surgery. Additionally, investigations of veCAP responses in human trials showed that these responses often fail to correlate well with postoperative measurements [56, 68]. Therefore, techniques to optimize electrode placement are still desired to facilitate optimal stimulation.

### Hearing Preservation

Bilateral vestibulopathy often occurs along with (severe) hearing loss. Patients with bilateral vestibulopathy who have an indication for a cochlear implant can benefit from a combined vestibular and cochlear implant. These combined implants were developed to aid these patients and to prevent adverse impact on hearing of only vestibular implantation in patients with residual hearing. However, only a third of the patients have a profound hearing loss in their worse hearing ear, thus are cochlear implant candidates. Even approximately half of the patients have normal hearing or a moderate hearing loss in both ears [3]. Consequently, many patients that could benefit from vestibular implantation would benefit from a technique with hearing preservation.

The exact impact of vestibular implantation on hearing, especially with the intralabyrinthine surgical technique, is not clear yet. In the extralabyrinthine technique [30, 45], there is a small risk of sensorineural hearing loss, possibly related to potential exposure of the perilymphatic compartment [69]. Additionally, this approach involves temporarily removing auditory ossicle(s) to reach all ampullary nerve canals, which may lead to a slight conductive hearing loss as well. In the intralabyrinthine technique, the inner ear is opened in order to insert the electrodes in the semicircular canal ampullae. Consequently, this involves opening the perilymphatic space and possibly also the endolymphatic space, when the membranous labyrinth is perforated. In a case report with only intraoperative hearing measurements using auditory brainstem responses, no hearing deterioration was detected during the surgical implantation procedure, as auditory brainstem response morphology remained stable and wave V was consistently detected after insertion and removal of an electrode dummy in the lateral and posterior semicircular canal (the superior canal was not implanted) [70]. Intralabyrinthine implantation in four patients with preexisting moderate or severe sensorineural hearing loss attributed to Menière’s disease resulted in profound sensorineural hearing loss with pure tone thresholds > 90 dB HL [56, 68]. However, vestibular implantation with subsequent continuous stimulation in patients with aminoglycoside-induced (*n* = 7) or idiopathic bilateral vestibulopathy (*n* = 1) and normal preoperative hearing at some or all frequencies had variable impact on hearing during 6 months follow-up. In one patient with a pre-existing high-frequency sensorineural hearing loss pure tone detection thresholds remained within 5 dB HL of preoperative values, while three patients experienced a high-frequency hearing loss, one had a ~ 30 to 35 dB HL change in pure tone thresholds across all frequencies, and three suffered loss to ≥ 80 dB HL across all frequencies [53]. The mechanisms behind hearing loss after intralabyrinthine vestibular implantation and inter-subject differences are not clear yet. It might be related to opening of the perilymphatic and/or endolymphatic compartments. The latter might not necessarily be opened in a vestibular implantation procedure. Additionally, insertion of the electrode lead might cause destructive pressure changes in the cochlear compartment. Currently, an ongoing trial investigates hearing evolution after each surgical step to investigate the contribution of each step to hearing deterioration (Vestibular implantation and inner ear preservation, International Clinical Trial Registry Platform (ICTRP) ID: NTR7017). Besides these surgical steps, hearing deterioration might also occur during postoperative follow-up [53], e.g., due to local inflammation. Furthermore, electrical stimulation of a combined vestibular and cochlear implant might lead to cross-stimulation and temporarily impact hearing during active use [61].

The majority of potential beneficiaries of a vestibular implant have good hearing; consequently, it would not require a combined vestibular and cochlear implant. Hence, when vestibular implantation with hearing preservation becomes possible, this would considerably improve the potential individual and societal impact of a clinically available vestibular implant.

### Labyrinth Preservation

**Fig. 2:**
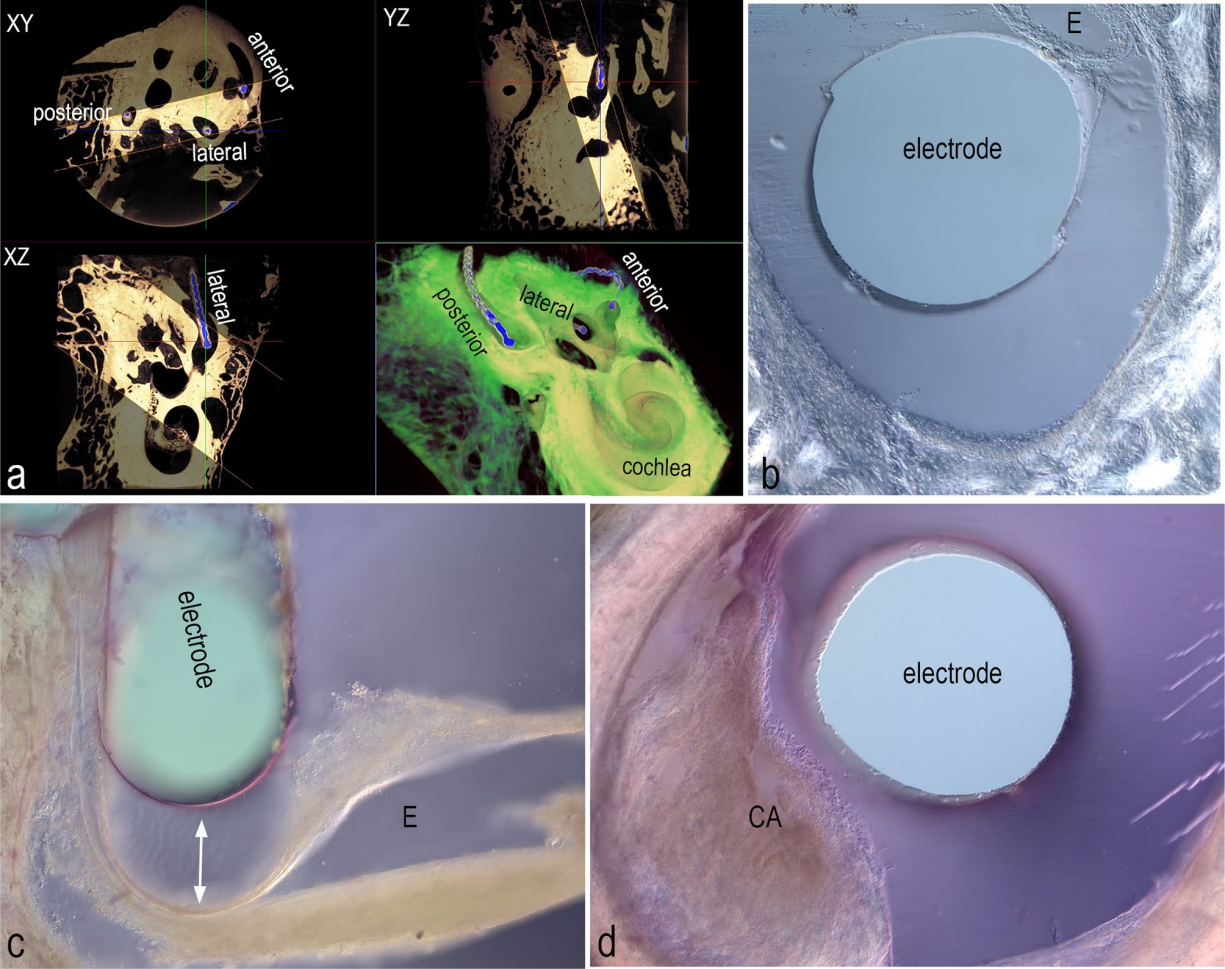
**a** Micro-CT imaging of an ossified metal artifact corrected implanted human inner ear; xy, -yz and -xz planes are resolved at 20 µm/voxel; lateral, anterior, and posterior semicircular canal with metal wires of vestibular electrodes are segmented blue; crosshair intersection represents the tip of the lateral canal electrode; highlighted slice within orange lines represents the 3D view in the lower right image; **b** vibratome section through a lateral semicircular canal with the electrode position preserved as an empty space in the gelatin matrix: the endolymphatic compartment (E) is completely preserved; **c** histological section of an over-inserted electrode in the posterior canal close to an ampullar organ: retraction (double arrow) of the electrode ending is evident (E-endolymph); **d** over-inserted electrode showed severe damage of the crista ampullaris (CA) sensorineural epithelium exposing ruptures and compressions

Vestibular electrode design aims to get closest to sensorineural structures while causing minimum damage to the labyrinth. After all, this might induce hearing loss and loss of residual vestibular function. Additionally, it might possibly decrease functioning of the implant. The impact of vestibular electrode insertion was tested in a cadaveric set-up utilizing micro-CT and correlative histology. Prototype vestibular implant electrodes (Med-El, Innsbruck, Austria) were inserted in fresh frozen cadaveric heads using an intralabyrinthine approach. Each semicircular canal was fenestrated away from the ampulla and fluoroscopy was used to guide the electrode insertion to the ampulla. Subsequently, the electrodes were fixated and the inner ear was extracted. Assessment of electrode position and possible tissue trauma was tested in a dual approach using contrast enhanced micro-CT scanning and correlative histology generating rather thick vibratome serial sections. Osmium tetroxide integrates into unsaturated fatty acids and thereby aggregates in all membranes. After temporal bone decalcification, the heavy metal osmium provides excellent contrast for low energy x-ray imaging (45kVp) and especially accentuates myelinated nerve fiber fascicles [71]. This technique not only distinguishes nerve fiber tracks through the entire inner ear but allows to visualize the exact position of the barium sulfate contrast–enhanced electrode silicone, metal components as well as sensory epithelia at high resolution (voxel size 10 µm). Metal scanning artifacts could be suppressed successfully in high energy scans (70kVp) before decalcification on the cost of resolution (Fig. 2a) but cannot entirely be eliminated in decalcified specimens with low energy scanning. To overcome these “blind spots” and get maximum details, temporal bones were embedded in gelatin, postfixed in formalin and cut with a vibratome at 100 µm thick slices (Fig. 2b–d). Hematoxylin–eosin staining with differential interference contrast imaging showed us that the endolymphatic membranes are completely preserved (Fig. 2b) but partially quite compressed by the electrode. Histological examination exposes severe damage of the sensory epithelium caused by overinsertion (Fig. 2d), visible even when the electrode was retracted (Fig. 2c). The gelatin matrix enabled safe detection of the electrode position even if the inorganic material gets lost during sectioning. 3D micro-CT imaging provided valuable data on electrode position and localization (Fig. 2a) in relation to sensory structures and nerve fiber pathways while histology added resolution and contrast to detect even the slightest damage of delicate membranes and ampullar organs. Data may further be valuable for computational models of vestibular electrostimulation, fluid simulation, and possible cochlear nerve cross-excitation in real human inner ears.

### Bringing the Implant to the Clinic

Bringing vestibular implantation into widespread use as a treatment for bilateral vestibulopathy will require regulatory approval, gaining approval from governmental or private third-party payers, and establishing a clinical care infrastructure similar to what already exists for delivery of clinical care related to cochlear implantation.

Standards for medical device approval vary by country and regulatory pathway, but all countries and pathways require data sufficient to determine that the device will not expose patients to an unreasonable risk and that the probable health benefit of using the device outweighs the risk of injury or illness from its use. The combined world experience of > 30 vestibular and vestibular combined with cochlear implantations has already demonstrated feasibility and safety of vestibular implant surgery with regard to general health. Measurement of reflexive eye movements and postural responses to modulated electrical pulses during brief test sessions in a laboratory or research clinic setting demonstrated that prosthetic stimulation can elicit movement sensation and drive vestibular reflexes. Surgical implantation was also shown to be feasible in an outpatient setting when no absolute or relative contraindications, such as comorbidity, postoperative dizziness, or patient preference, were present. However, garnering regulatory approval of a vestibular implant system intended for use at home as a sensory restoration prosthesis will require data sufficient to clarify risk and benefit after long-term, continuous use of vestibular implant systems at home. Additionally, socioeconomic impact should be demonstrated. A cost-utility analysis suggested favorable cost-effectiveness of a vestibular implant: considering 50–100% vestibular function restoration, a cost-utility of $28,490–$56,979 per quality-adjusted life-year was shown [2].

A currently ongoing trial investigates continuous home use of a vestibular implant (Multichannel Vestibular Implant Early Feasibility Study, ClinicalTrials.gov Identifier: NCT02725463). As of February 2021, eight patients had undergone unilateral implantation and continuous (either 24 h/day or during all waking hours) motion-modulated prosthetic stimulation during at least 6 months of follow-up [33, 53]. Measures of posture, gait, and quality of life improved or were in the direction of improvement from baseline. Various deterioration of hearing was, however, shown in seven patients, as described above. Other reported adverse events include falls, tinnitus, transient imbalance, dysgeusia, facial twitch, and tingling that stopped when the stimulus current was reduced. Two participants reported in total three falls while having the device turned on: two falls in one patient were related to a software error that led to sudden discontinuations of stimulation and one patient fell and fractured his clavicle during cycling. Regarding patient-reported outcomes, scores on the Dizziness Handicap Inventory and on the health utility index derived from the 36-Item Short-Form Health Survey improved significantly after 6 months and 1 year after implantation, while others where in the direction of improvement. Overall, the results of this trial support the conclusion that implantation and long-term use of a vestibular implant will not expose patients to an unreasonable risk and that the probable health benefit of using a vestibular implant outweighs the risk of injury or illness from its use.

The current vestibular implant prototypes were aimed to aid patients with bilateral vestibulopathy, but a vestibular implant can potentially also become helpful for patients with other vestibular conditions, such as unilateral vestibular hypofunction or conditions with fluctuating vestibular function, in which the implant interacts with residual function as a “vestibular pacemaker” [32]. In 2020, an opinion statement was published that defined criteria for vestibular implantation in current research setting [72]. These criteria include a combination of patient-reported (disabling) symptoms and objective criteria regarding bilateral vestibulopathy, including bilaterally reduced or absent angular VOR function documented by video Head Impulse Test or scleral-coil technique, caloric testing, and sinusoidal stimulation on a rotatory chair. Additionally, general requirements for initial preclinical trials are stated. Ongoing and future (pre)clinical trials will presumably provide additional data for potential refinement of the criteria for clinical trials and eventually, clinical practice.

## Conclusion

By extensive research in animals and humans, using different prototypes and different approaches, the concept of a vestibular neuroprosthesis has been demonstrated. Taking the results of these investigations into account, it seems to become a clinically useful device in the near future. Still, challenges remain. Next challenges to overcome moving forward in the development of a clinically applicable vestibular implant are discussed in this article, although the challenges are undoubtedly not limited to the ones mentioned.

At first, optimizing the electrical stimulation parameters will improve the dynamic range, the selectivity and the end organ responses such as VOR. This should take into account the electrode design and the location of the inserted electrode, as these combinations will influence the selectivity of targeted nerve stimulation, and in order to limit undesired current spread. The surgical implantation procedure, and related the electrode design, may be improved so that the stimulating electrodes can be implanted with more precision to improve the stimulation, as well as with minimal damage to the labyrinth to preserve residual vestibular and cochlear function. Imaging techniques and/or biophysiological measurements might be needed to achieve these goals. Finally, implanted prostheses should be tested in ambulatory care settings. Subsequently, approval from regulatory authorities and governmental or third-party payers should be obtained and a clinical care infrastructure should be set up to provide postoperative fitting and possibly (ambulatory) rehabilitation care for these patients.

Even though there will always be room for progress, as long as regenerative therapies come up short, finding solutions to these problems could pave the way for the realization of a clinically applicable vestibular implant that can significantly benefit a large number of patients.

This article outlines and elaborates on what was presented by the authors and discussed during the identically named symposium at the 44th Annual MidWinter Meeting of the Association for Research in Otolaryngology in 2021. The displayed discussions are built on the research done by several research groups. Investigations with different approaches and different vestibular implant prototypes resulted in complementary, mutually reinforcing and sometimes reproduced results. These observations strengthen the concept of the development.

The authors look forward to collectively advance in this emerging field of research and development and confer progress with fellow JARO readers and during future meetings. Based on the research conducted by various research groups throughout the world, we hope to create a clinically applied device in the near future.
